# Evaluating the accuracy and reliability of AI chatbots in disseminating the content of current resuscitation guidelines: a comparative analysis between the ERC 2021 guidelines and both ChatGPTs 3.5 and 4

**DOI:** 10.1186/s13049-024-01266-2

**Published:** 2024-09-26

**Authors:** Stefanie Beck, Manuel Kuhner, Markus Haar, Anne Daubmann, Martin Semmann, Stefan Kluge

**Affiliations:** 1 Department of Intensive Care Medicine, Hamburg-Eppendorf University Medical Centre, Hamburg, Germany; 2 Department of Medical Biometry and Epidemiology, Hamburg–Eppendorf University Medical Centre, Hamburg, Germany; 3 Hub of Computing and Data Science, University of Hamburg, Hamburg, Germany

## Abstract

**Aim of the study:**

Artificial intelligence (AI) chatbots are established as tools for answering medical questions worldwide. Healthcare trainees are increasingly using this cutting-edge technology, although its reliability and accuracy in the context of healthcare remain uncertain. This study evaluated the suitability of Chat-GPT versions 3.5 and 4 for healthcare professionals seeking up-to-date evidence and recommendations for resuscitation by comparing the key messages of the resuscitation guidelines, which methodically set the gold standard of current evidence and recommendations, with the statements of the AI chatbots on this topic.

**Methods:**

This prospective comparative content analysis was conducted between the 2021 European Resuscitation Council (ERC) guidelines and the responses of two freely available ChatGPT versions (ChatGPT-3.5 and the Bing version of the ChatGPT-4) to questions about the key messages of clinically relevant ERC guideline chapters for adults. (1) The content analysis was performed bidirectionally by independent raters. The completeness and actuality of the AI output were assessed by comparing the key message with the AI-generated statements. (2) The conformity of the AI output was evaluated by comparing the statements of the two ChatGPT versions with the content of the ERC guidelines.

**Results:**

In response to inquiries about the five chapters, ChatGPT-3.5 generated a total of 60 statements, whereas ChatGPT-4 produced 32 statements. ChatGPT-3.5 did not address 123 key messages, and ChatGPT-4 did not address 132 of the 172 key messages of the ERC guideline chapters. A total of 77% of the ChatGPT-3.5 statements and 84% of the ChatGPT-4 statements were fully in line with the ERC guidelines. The main reason for nonconformity was superficial and incorrect AI statements. The interrater reliability between the two raters, measured by Cohen’s kappa, was greater for ChatGPT-4 (0.56 for completeness and 0.76 for conformity analysis) than for ChatGPT-3.5 (0.48 for completeness and 0.36 for conformity).

**Conclusion:**

We advise healthcare professionals not to rely solely on the tested AI-based chatbots to keep up to date with the latest evidence, as the relevant texts for the task were not part of the training texts of the underlying LLMs, and the lack of conceptual understanding of AI carries a high risk of spreading misconceptions. Original publications should always be considered for comprehensive understanding.

## Introduction

Artificial intelligence (AI) driven chatbots, such as ChatGPT, have garnered attention by successfully passing the U.S. Medical Licensing Examination (USMLE) and knowledge assessments in Basic life support (BLS) and Advanced life support (ALS) [2–6]. Numerous publications underscore the vast potential these technologies hold for enhancing patient care, augmenting diagnostic capabilities, and shaping the future landscape of medicine [7–10] . Technical journals highlight the capacity of ChatGPT and its prospective role as a clinical decision aid, offering real-time, evidence-based recommendations to healthcare practitioners [10, 11].

Despite the immense potential of AI-based chatbots, these models have failed to provide appropriate advice, particularly in clinical situations that involve ethical and emergency dimensions [12, 13]. To accurately gauge the potential benefits and risks associated with employing AI as an advisor for healthcare providers, a comprehensive understanding of the underlying architecture and operational methodology is crucial [14, 15].

AI-based chatbots such as ChatGPT are powered by a large language model (LLM) that uses deep learning techniques to produce human-like responses with new content to natural language inputs. The LLM architecture of ChatGPT is based on the transformer architecture, which is composed of several components, such as multi head attention, feedforward neural networks, and residual connections [16]. These components work together to enable the model to generate responses. (For a comprehensive explanation of the LLM architeture we refer this source: [17].

The generation process can be explained according to ChatGPT-4 with these four steps:

1. **Training**: ChatGPT is trained on a large amount of text data. During this training, it learns to predict the next word in a sentence. It does this millions of times and, in the process, identifies patterns such as basic grammar and seemingly factual data on the basis of the training data.
2. **Understanding the Prompt**: When you type a message, ChatGPT uses what it learned during training to understand the prompt. It does not understand the message like a human would. Instead, it processes the sequence of words and generates a mathematical representation for them.
3. **Generating a Response:** To generate a response, ChatGPT considers many possible next words and chooses the one that it assesses to be the likeliest, on the basis of what it learned during training. This process is repeated for each word in the response until a complete message is generated.
4. **Optimisation**: The responses are also optimised to be aligned with the assistant’s guidelines. This includes refusing certain types of requests, avoiding controversial topics, etc. Those guardrails are not transparent and do not necessarily meet the legal obligations in every given country and do not need to meet ethical standards.

Consequently, the knowledgebase of Chat-GPT is contingent upon the timeliness and accuracy of its training data. For instance, GPT-3.5’s knowledge is constrained as it is founded on information available only up until September 2021. The generation process indicates that the generative AI model produces the statistically most likely response to a given prompt, which might not always be factually correct. This issue, known as ‘hallucination’ in Large Language Models (LLMs), pertains to errors in the generated text that appear semantically or syntactically plausible but lack factual or evidential basis [18, 19]. Given that the output of generative AIs, is typically challenging to verify, considerable effort has been invested in the optimisation process.

In order to drive optimisation and ensure consistently high-quality responses, OpenAI employs a combination of both supervised learning and reinforcement learning with human feedback (RLHF) [20]. Supervised learning involves training the model on a vast corpus of text data with labelled examples of inputs and outputs. The model is trained to predict the output given the input, and the weights of the model are adjusted to minimise the discrepancy between the predicted output and the actual output. On the other hand, RLHF involves training the model to maximise a reward signal on the basis of its actions. The evaluation of the response quality by a human evaluator helps refine the model’s responses in an iterative process over time [21].

While optimisation has significantly improved performance, especially in correctly answering knowledge tests, the process of generating responses carries a substantial risk of misinformation [22].

Evaluating whether ChatGPT is a suitable tool for healthcare professionals with a clinical focus, such as medical students, physicians, nurses, and EMS personnel, to keep up to date with the latest developments and advancements in resuscitation is interesting for three reasons.

First, the increasing use of AI chatbots, especially by students under the age of 35 years, and the underdeveloped research and evidence-based practice skills in this age group require scientific studies to advise both AI chatbot users and healthcare professional educators on the risks and benefits [23–25].

Second, with a focus on the purpose of the international nonprofit association ILCOR ‘to promote, disseminate and advocate international implementation of evidence-informed resuscitation and first aid, using transparent evaluation and a consensus summary of scientific data’, AI chatbots have great potential to support dissemination by removing language barriers and providing free access. ILCOR's mission to disseminate evidence in low-resource settings could be facilitated by the elimination of translation work [23].

Third, it is not publicly known which texts have been used for ChatGPT training. The clear wording, official publication date and special focus of the guidelines every four years make it methodologically feasible to determine whether the current guideline text and the underlying primary literature were part of the training text of the AI chatbots.

Therefore, this study investigated not only whether Chat-GPT-generated output is in line with the content of the European Resuscitation Council (ERC) guidelines but also whether ChatGPT can accurately, reliably and completely reproduce the content of the key messages of the current ERC guidelines.

## Methods

### Study design and AI outputs

We conducted a prospective comparative content analysis between the content of the ERC guidelines published in March 2021 and the responses of two freely available versions of ChatGPT (ChatGPT-3.5 and the Bing version of ChatGPT-4) when we asked about the key messages of the ERC chapters [1].

The analysis was centred on content pertinent to patient treatment and encompassed the chapters on Basic life support (BLS), Advanced life support (ALS), special circumstances, post-resuscitation care, and ethics.

To generate responses, the AI was prompted with the following question: “What are the key messages of the chapter ‘xx’ of the ERC guidelines 2021?” This approach generated a list of statements for every chapter, was performed with both Chat-GPT-Versions and allowed us to directly compare the AI’s understanding and reproduction of the guidelines with the original text and the key messages files.

The closed-source AI, Chat-GPT 3.5, was accessed as free to use version on July 12, 2023. The complimentary Bing version of Chat-GPT 4 was subsequently accessed on September 5, 2023. All prompts pertaining to the relevant chapters were posed within a single session in the sequence in which they appeared in the guidelines.

### Content analysis

A practice test (piloting) was performed with two guideline chapters not intended to be analysed (Newborn resuscitation and Paediatric life support). The comparative analysis of the key messages of the guidelines and the AI-generated statements revealed a discordance between the key messages and the statements in terms of number and content. Based on the surprisingly much lower number of statements generated by AI compared to key messages of the ERC, we decided two perform a bidirectional comparative content analysis by two independent raters.

#### Analysis of completeness

The completeness and actuality of the AI output were assessed by comparing every key message of the 5 chapters, which were published concurrently with the ERC chapters and accessed on July 12, 2023, with the statements generated by the AIs. The analysis is called “completeness and actuality” because, due to the black-box nature of the LLM, it is not possible to distinguish whether the training texts were too old, and the content was ‘not yet’ integrated or whether the selection of training texts was not suitable to reflect the full breadth of knowledge. Methodologically, we can therefore only measure completeness, but we should bear in mind that this could also be a consequence of the lack of actuality. The completeness was rated via a three-point ordinal scale with the categories “completely addressed”, “partially addressed”, and “not addressed by the AI”. If a sentence received a “partial” rating, specific error types were identified. Drawing from the literature, we anticipated the following error types: “superficial” (the statement lacks sufficient detail for full application), “inaccurate” (certain elements of the statement are not applicable), and “hallucination” (the statement appears credible but is unsupported by evidence) [18, 24]. During the analysis, the raters introduced an additional error type, “failure to distinguish between evidence and recommendation”, for the comparison between the key messages and the AI output.

#### Analysis of conformity

The conformity of the AI output was evaluated by comparing the output of Chat-GPT with the content of the ERC guidelines. Therefore, we checked for every AI-generated statement if there was a corresponding statement in the key messages of the chapter. If not, we consulted the full text of the guideline chapter and searched for a corresponding statement. Conformity was rated via a three-point ordinal scale with the categories “completely conform”, “partially conform”, and “not conform with the guidelines”. For “partial” ratings, specific error types were identified. In addition to the literature-based error types: “superficial,” “inaccurate,” and “hallucination,” the error type “not addressed in this chapter/guideline version” was added for the comparison between the AI output and the guidelines.

#### Rater agreement

After the initial independent rating by the two raters (SB and MK), who are physicians specialised in intensive care medicine and teaching in resuscitation including ERC courses, discordant ratings were discussed, and a consensus was reached for the final rating. For the four content analyses (completeness and conformity for both Chat-GPT versions), the interrater agreement was calculated via Cohen’s kappa.

#### Statistical analysis

Descriptive statistics were performed with SPSS (version 24, IBM Corp., Armonk, New York, USA). Absolute and relative frequencies were calculated for the categorical variables. Cohen’s kappa was used to calculate the interrater agreement.

## Results

In response to inquiries about the five chapters, ChatGPT-3.5 generated a total of 60 statements, whereas ChatGPT-4 produced 32. The number of statements generated by the AIs was fewer than the number of key messages for each chapter. In total, 172 key messages were compared with the AI outputs (Table 1).

**Table 1:** Number of messages addressing the different guideline chapters

Chapter	ChatGPT-3.5	ChatGPT-4 (Bing)	Key messages
Basic life support (No.)	12	8	27
Advanced life support (No.)	15	10	25
Special circumstances (No.)	11	5	44
Post-resuscitation care (No.)	14	4	29
Ethics (No.)	8	5	47
Total (No.)	60	32	172

### Completeness and actuality of ChatGPT

When asked about the key messages, ChatGPT-3.5 provided a reference to the official ERC website for accurate and up-to-date information, noting that its knowledge cut-off was in September 2021. ChatGPT-4, on the other hand, began its response with a brief summary of the chapter without mentioning any access restrictions to the guideline text.

**Fig. 1:**
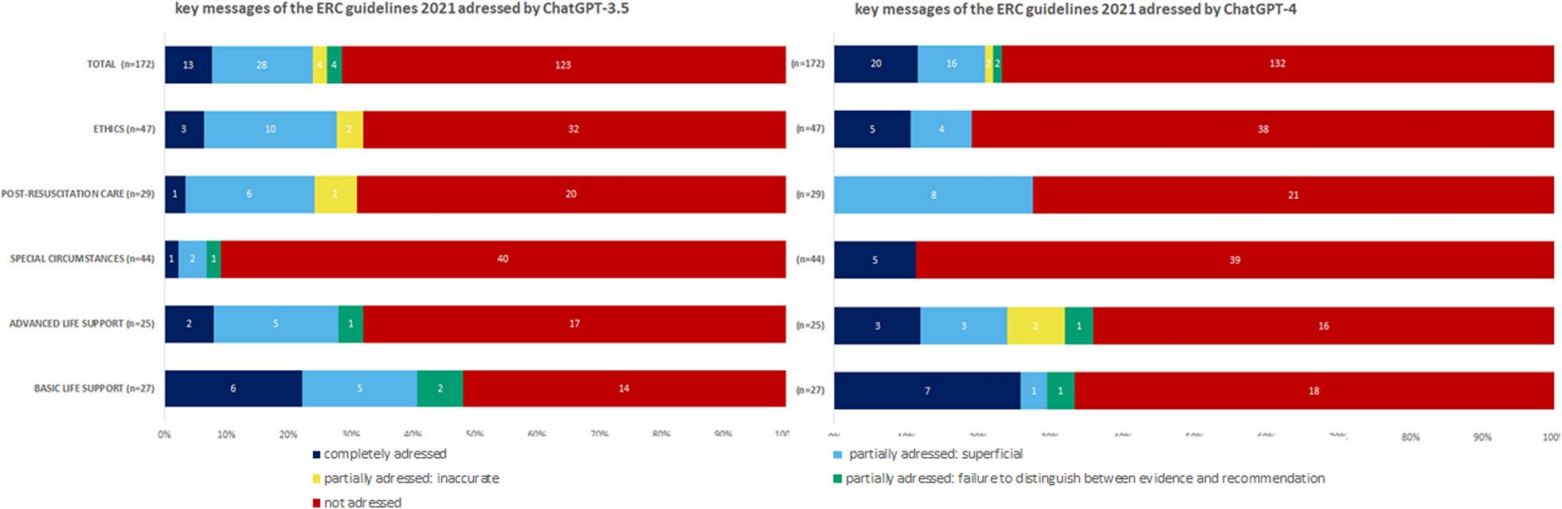
Results of the performance analysis of two ChatGPT versions in addressing the key messages of clinically relevant ERC guideline chapters

Among the 172 key messages, ChatGPT-3.5 addressed 13 key messages completely and failed to address 123, whereas ChatGPT-4 addressed 20 key messages completely and did not address 132. Both versions of ChatGPT more frequently addressed BLS key messages completely than they did key messages from other chapters. In all the other chapters, more than two-thirds of the key messages were not addressed at all (Fig. 1).

ChatGPT-3.5 partially addressed 36 key messages, whereas ChatGPT-4 partially addressed 20. The error type “superficial” was assigned 28 times for ChatGPT-3.5 and 16 times for ChatGPT-4. The error “inaccurate” was noted four times in ChatGPT-3.5 and twice in ChatGPT-4. In ChatGPT-3.5, four sentences, and in ChatGPT-4, two sentences did not distinguish between evidence and recommendation (Fig. 1).

#### Examples for the error type “superficial”

Key message on post-resuscitation care: “Use multimodal neurological prognostication using clinical examination, electrophysiology, biomarkers, and imaging”.

Corresponding ChatGPT-4 statement: “Post-resuscitation care should include early coronary angiography and revascularization when indicated, targeted temperature management, seizure control, multimodal prognostication, and organ donation when appropriate.”

#### Example for “not discriminating between evidence and recommendation

Key message on BLS: “AEDs can be used safely by bystanders and first responders”.

Corresponding ChatGPT-3.5 BLS statement: “Public access defibrillation: Encourage the use of public access defibrillation programs to improve early defibrillation in the community.” “Use of AEDs: Promptly apply an AED when available, ensuring the correct pad placement and following voice and visual prompts.”

#### Conformity

#### ChatGPT-3.5

**Fig. 2:**
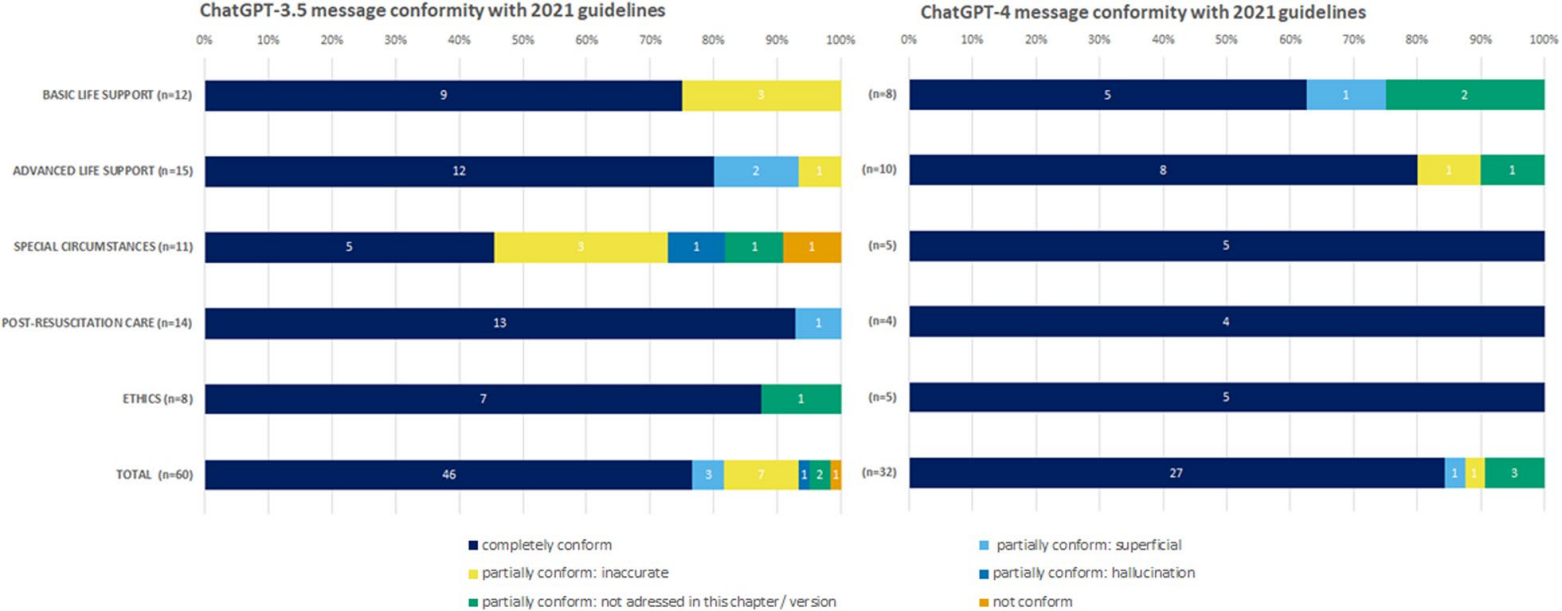
Results of the performance analysis of two ChatGPT versions for the accordance of the AI output with the ERC guideline text for the relevant chapters

Of the 60 output statements of ChatGPT-3.5, 46 (77%) were in accordance with the guidelines. Thirteen statements were partially conform with the guidelines and these statements were distributed across all the chapters. In the chapter on special circumstances, one sentences was not conform with the guidelines because it addressed a symptom (burns) which is associated with cardiac arrest due to electrocution and lightning strike but is outside the scope of the resuscitation guidelines (Fig. 2). Among the 13 statements rated as partially conform with the guidelines, three messages were deemed too “superficial,” seven were “inaccurate,” one was “hallucinated,” and two were “not addressed in this chapter or guideline version.” (Fig. 2).

An example of an inaccurate statement is “Begin chest compressions as soon as possible in a ratio of 30 compressions to 2 rescue breaths for adult and pediatric patients.” (ChatGPT-3.5, Chapter BLS).

A comparison of the statement with the key messages from 2021 on BLS of the ERC guidelines page (ERC Guidelines cprguidelines.eu) demonstrated that this recommendation only applies to adults.

An example of a hallucinated statement is “Initiate CPR immediately, and once the patient is in a hospital setting, consider rewarming and further management of potential complications such as pulmonary edema or hypoxemia.” (ChatGPT-3.5, Chapter special circumstances).

Comparing the statement with the key messages from 2021 on Special Circumstances (ERC Guidelines cprguidelines.eu) demonstrates that this recommendation is not part of the guideline text and that hypoxia and pulmonary edema must be addressed immediately. The temporal separation is hallucinated.

#### ChatGPT4

Out of the 32 output statements from ChatGPT-4, 27 (84%) were conform with the guidelines. For BLS and ALS, some statements were only partially conformed with the guidelines (Fig. 1). One statement was deemed too “superficial,” one was “inaccurate,” and three were “not addressed in this chapter/guideline version.”

An example of a superficial statement (because the clinical condition of the patient is not respected): “Foreign body airway obstruction should be managed by encouraging the victim to cough, followed by back blows and chest thrusts (or abdominal thrusts in obese or pregnant victims).” (ChatGPT-4, Chapter BLS).

An example of a message “not addressed in this guideline version”: “Minimizing interruptions in chest compressions and avoiding excessive ventilation are essential to optimize blood flow and oxygen delivery during CPR.” (ChatGPT-4, Chapter ALS).

### Rater agreement

The analyses of the interrater agreement revealed for the comparison of the key messages with the AI statements (completeness analysis) a moderate interrater reliability for both versions (Cohen’s kappa: 0.48 for ChatGPT-3.5 and 0.56 for ChatGPT-4).

In terms of the conformity of the AI output with the guidelines (conformity analysis) the interrater reliability, as measured by Cohen’s kappa, was significantly better for ChatGPT-4 (0.76) than for ChatGPT-3.5 (0.36).

## Discussion

Based on the results of our performance evaluation of Chat-GPT against the latest ERC guidelines, we found that Chat-GPT did not completely report the content of the key messages (failing to address two-thirds of the key messages), but the generated AI output was conforming to the guidelines in 77% (Chat-GPT-3.5) and 84% (Chat-GPT-4), respectively. Therefore, the sole use of ChatGPT as an accurate and reliable information resource for healthcare professionals seems insufficient. To acknowledge all the results and provide a more nuanced response, we have divided the discussion into three parts. First, we recognise the performance of AI as an information source. Second, we examine the tool’s appropriateness for healthcare professionals. Finally, we discuss the strengths and limitations of this study to achieve a precise and balanced conclusion.

### Performance as an information source

ChatGPT-3.5 clearly indicated its limitations as an information source, noting that its knowledge was based on information available until September 2021. It recommended referring to the latest ERC guidelines for the most accurate and up-to-date information, whereas the bing version of ChatGPT-4 did not explicitly draw the user’s attention to its limitations.

Despite the general nature of the inquiries on the key messages of the ERC guideline chapters, the AI was able to maintain focus. Only one statement from ChatGPT-3.5 was not related to resuscitation. The high conformity of 77% (ChatGPT-3.5) and 84% (ChatGPT-4) of the AI statements with the guidelines suggests a certain ability of the generative AIs to summarise and reproduce medical knowledge accurately. This is in line with previous studies that reported high scores for AI-based chatbots in tests inquiring about medical knowledge [2, 4, 5].

As anticipated, both versions of the Chatbot were prone to errors and produced incorrect and superficial responses. Surprisingly, ChatGPT-3.5 generated only one piece of false information, which seemed plausible but did not conform to the guidelines. The hallucinated sequence of treatment steps it suggested would be ineffective. The clinical significance of these errors varies depending on the target audience. For laypeople with limited knowledge and experience, the harm may be limited. The benefit from increased basic information likely outweighs the risk of prompting suboptimal bystander actions. Notably, the AI performed worse in the chapters of broader interest (BLS and ALS) than in the more specialised chapters.

### Suitability for healthcare professionals and experts

While producing less output, ChatGPT-4 was more in line with the guidelines, but it addressed fewer key messages, both completely and partially. The interrater agreement concurrently improved from fair to moderate from ChatGPT versions 3 to 4, according to the scale of Landis and Koch [25].

The enhanced performance of version 4 compared to version 3.5. in medical question-answering and increased reliability through clearer phrasing align with the test results published in ChatGPT-4’s technical report [26] However, the reliability among raters is still far from optimal or satisfying. Raters found it challenging to determine whether the AI’s altered wording still accurately represents the statements’ underlying causal and conditional relationships. This challenge for the raters may arise from the nature of the LLM function, which represents a statistical understanding of training data but lacks the conceptual understanding to genuinely comprehend real-world phenomena. The LLM's lack of conceptual understanding is consistent with studies reporting insufficient semantic sensitivity and ability to understand relationships and contrasts strongly with the nature of experts' information needs [27, 28] For professionals who fulfil the role of a medical expert (as defined by the CanMed Roles), adequate patient care requires close adherence to the evolving body of knowledge [29] However, we do not recommend using AI chatbots when focusing on understanding the topic, as the lack of conceptual understanding of LLM makes it even more difficult to understand the context, objectives, and limitations of evidence.

Our observation of the failure of AIs to address two-thirds of the key messages is in line with studies that highlight the limited ability of LLMs to conform with the current scientific consensus [9] Although the official knowledge cut-off of ChatGPT version 3 was September 2021, the LLM was not trained with the guideline text and other texts that reflect the clinical topics that were important at that time.

The performance of LLMs in reporting specific content can be improved by using LLMs that allow real time internet searches or that can be provided with documents allowing data extraction from reliable (known) sources (e.g. ChatGPT [OpenAI], Perplexity [https://www.perplexity.ai], Gemini [Google]). From a technical perspective, it is unclear whether the general inclusion of guideline texts in the corpus of training texts can be influenced (given that AI chatbots are owned by private companies), and whether the intellectual input, provided by experts during evidence discovery and the justification for the recommendation, is reflected in the AI’s output. Retrieval-augmented generation (RAG) may be helpful to provide LLMs with the data they need to develop conceptual understanding and to improve accuracy and reliability of the generation process. RAG is an AI framework, which grounds the model on external sources of knowledge to supplement the LLM's internal representation of information [30, 31].

### Strengths

The strengths of this study, which was one of the first to investigate the potential benefits and suitability of open-access AI chatbots as a source of information for healthcare providers, are its methodological quality and real-world utility. Using the key messages as the gold standard of current knowledge and clinical reasoning in resuscitation ensured the methodological quality in two ways and enabled a quantitative measurement. First, the key messages are the best reference in terms of both content and language in regard to current evidence and recommendations in resuscitation, on the basis of their methodical rigour of development. Second, the keyword-like formulation allows a direct comparison with the output of the AI chatbots 1:1.

Piloting the comparative analysis of the AI statements with the key messages of other chapters revealed that the number and focus of the statements (AI outputs) differed greatly from those of the key messages and overlapped. This challenge was addressed by a bidirectional analysis, which allowed us to assess the potential harm of following the AI statements (conformity between the statements and the key messages) and the use of AI chatbots for healthcare providers to stay informed about current evidence and recommendations by comparing the key messages as the gold standard with the AI statements (completeness and actuality).

By integrating a research business information scientist into the research team, we were able to ensure that the assessment, whether the phenomena were due to information technology or the user, was well founded and based on scientific expertise in IT.

By measuring and reporting interrater reliability, a quality indicator of comparative studies, we inadvertently discovered that poor interrater reliability might indicate insufficient training of the LLM on the topic. This hypothesis is supported by studies that achieved higher reliability and validity in the generation process by using methods that effectively ground LLM representations in external sources of knowledge [30, 32].

## Nevertheless, we must admit some important limitations of our study

### Limitations

#### Generalizability

The findings from AI chatbots have limited generalizability because of their dynamic nature. The output from these chatbots is influenced by several factors, including the phrasing of questions, the user’s previous interactions with the AI, and ongoing optimisation processes conducted by the providers. As such, repeating the study may not yield identical results.

#### Specificity to the bing version

This study was conducted via the Bing version of the GPT-4, which has been specifically tuned by Microsoft to better match their context. Therefore, the assessment of response quality cannot be generalised to the OpenAI version of the ChatGPT-4.

#### Reliability

The insufficient reliability between raters could also be partly due to differences in experience and general openness to/scepticism about technology. Further studies should systematically investigate rater characteristics and their influence on ratings.

#### Prompt stability

Unlike other performance measurement studies, we imitated the actual usage behaviour of inexperienced users. Therefore, we did not test the stability of the prompt. The prompt was sent only once in a single session rather than three times, which may affect the consistency of the results.

#### Relevance of key messages

The key messages were treated methodologically as equivalent, as there is no tool to compare the clinical relevance of the individual statements against each other, even if some statements appear more important than others.

#### Ethical considerations

This study did not investigate ethical considerations, which are relevant aspects of AI chatbot usage. ERC guidelines are subject to a more general ethical review than ChatGPT and all other Language learning models (LLMs). The origin of the model can impact its ethical performance. Furthermore, all LLMs face the challenge that the volume of training data required exceeds what can be ethically assessed.

## Conclusion

On the basis of our analysis, we can advise both AI chatbot users and educators of healthcare professionals on the risks and benefits of the tested AI chatbots. Owing to the lack of conceptual understanding, AI chatbots carry a high risk of disseminating misconceptions. The failure to reproduce a high percentage of the key messages indicates that the relevant text for the task was not part of the training texts of the underlying LLMs. Therefore, despite their theoretical potential, the tested AI chatbots are, for the moment, not helpful in supporting ILCOR's mission for the dissemination of current evidence, regardless of the user language. If integrating the recent guideline text into the training and retraining of language models (LLMs) could prove beneficial and if certain AI chatbots can assist healthcare professionals in locating relevant literature and extracting specific information, was not subject of this study. However, the active process of reception to understand a subject remains a fundamental prerequisite for developing expertise and making informed decisions in medicine. Therefore, all healthcare professionals should focus on literature supporting the understanding of the subject and refrain from trying to delegate this strenuous process to an AI.

## Data Availability

Relevant data are included within the body of this manuscript. All raw and analysed data and materials are securely held on a password protected computer system in the Department of Intensive Care Medicine of the University Hospital Hamburg-Eppendorf (where the study was completed). The datasets are not publicly available but are available from the corresponding author on reasonable request.

## Abbreviations

AI: Artificial intelligence
ALS: Advanced life support
BLS: Basic life support
ChatGPT: Chat generative pre-trained transformer
CPR: Cardiopulmonary resuscitation
EMS: Emergency medical services
ERC: European Resuscitation Council
LLM: Language learning model
RAG: Retrieval-augmented generation
RLHF: Reinforcement learning with human feedback
USMLE: U.S. Medical Licensing Examination
